# Construct, content and face validity of the eoSim laparoscopic simulator on advanced suturing tasks

**DOI:** 10.1007/s00464-018-06652-3

**Published:** 2019-01-22

**Authors:** Erik Leijte, Elke Arts, Bart Witteman, Jack Jakimowicz, Ivo De Blaauw, Sanne Botden

**Affiliations:** 1 Department of Paediatric Surgery, Radboud University Medical Centre - Amalia Children’s Hospital, Nijmegen, The Netherlands; 2 Department of Surgery, Rijnstate Hospital, Arnhem, The Netherlands; 3 Department of Industrial Design, Technical University Delft, Delft, The Netherlands; 4 Department of Surgery, Catharina Hospital, Eindhoven, The Netherlands

**Keywords:** Laparoscopy training, Simulation, Construct validity, Face validity, Content validity, Augmented reality simulator

## Abstract

**Background:**

The purpose of this study was to validate the eoSim, an affordable and mobile inanimate laparoscopic simulator with instrument tracking capabilities, regarding face, content and construct validity on complex suturing tasks.

**Methods:**

Participants recruited for this study were novices (no laparoscopic experience), target group for this training (surgical/gynaecologic/urologic residents, > 10 basic and < 20 advanced laparoscopic procedures) and experts (> 20 advanced laparoscopic procedures). Each participant performed the intracorporeal suturing exercise (Task 1), an upside down needle transfer (Task 2, developed for this study) and an anastomosis needle transfer (Task 3). Following, the participants completed a questionnaire regarding their demographics and opinion on the eoSim in terms of realism, didactic value and usability. Measured outcome parameters were time, distance, percentage of instrument tip off-screen, working area, speed, acceleration and smoothness.

**Results:**

In total, 104 participants completed the study, of which 60 novices, 31 residents and 13 experts. Face and content validity results showed a mean positive opinion on realism (3.9 Task 1, 3.6 Task 2 and 3.7 Task 3), didactic value (4.0, 3.4 and 3.7, respectively) and usability (4.2. 3.7 and 4.0, respectively). There were no significant differences in these outcomes between the specified expertise groups. Construct validity results showed significant differences between experts, target group or novices for Task 1 in terms of time (means 339, 607 and 1224 s, respectively, *p* < 0.001) and distance (means 8.1, 15.6 and 21.7 m, respectively, *p* < 0.001). Task 2 showed significant differences between groups regarding time (*p* < 0.001), distance (*p* 0.003), off-screen (*p* < 0.001) and working area (*p* < 0.001). Task 3 showed significant differences between groups, after subanalyses, on total number of stitches (*p* < 0.001), time per stitch (*p* < 0.001) and distance per stitch (*p* < 0.001).

**Conclusions:**

The results of this study indicate that the eoSim is a potential meaningful and valuable simulator in the training of suturing tasks.

The adoption of minimal invasive surgery as a frequently used surgical approach has had a significant impact on surgical practice. Traditionally, an important part of surgical training follows a master–apprentice model. This model is time-consuming and costly, and there is the risk of secondary harm to the patient due to trainee inexperience [1–4]. Also, the introduction of minimally invasive techniques requires a specific set of skills which creates a new teaching dynamic [4, 5]. Therefore, an alternative for this teaching model is the training of laparoscopic surgical skills in a simulated setting. Inanimate simulators are incorporated increasingly as a training module and a strong development of these systems is observed. However, this results in increasing costs with the advancement of the technology used [6]. Although, these expensive high-fidelity simulators are an interesting development they are not affordable to all and often lack mobility [7]. Therefore, there is still a need for more affordable simulators with the ability to give direct assessment to the trainee, to ensure a training, independent of the availability of expert observers. EoSurgical ltd. (Edinburgh, Scotland, United Kingdom) has recently developed the eoSim laparoscopic simulator, which is a relatively low-cost simulator ranging from £699 to £1298 at the time of writing, this includes the instrument tracking capability [8–11]. The main advantage of this system is that it is low in weight and easily transportable, to practice laparoscopic skills in any setting. It also shows performance parameters after each task while working with real instruments. Therefore, this simulator is an augmented reality simulator that allows parametric feedback independently of the task that is performed. This objective parametric feedback differentiates the eoSim from other affordable laparoscopic simulators without the instrument tracking capabilities.

Recent studies demonstrate a positive effect of pre-operative warm-up exercises on performance of the surgeon in terms of time and psychomotor abilities [12–16]. The eoSim could be an attractive tool to train laparoscopic psychomotor skills as a pre-operative warm up tool. Especially when used for more complex surgical procedures, such as suturing in small areas or complex angles (e.g. oesophageal anastomoses, jejunostomy placement). However, for a training system to be implemented in training programs, it has to be validated. Validation of a system in terms of face, content and construct validity is required to assess the systems value for training [17–19]. The aim of this study is to validate the eoSim laparoscopic simulator in terms of face, content and construct validity regarding complex suturing and needle transfer tasks.

## Method

### Participants

The participants were tested in a period of 5 months (February–June 2017) at the Radboud university medical centre Nijmegen, Rijnstate hospital Arnhem and Catharina hospital Eindhoven in the Netherlands. Additional participants were included during the ‘National Endoscopic Surgery symposium’, Amsterdam and the ‘National Surgeon’s congress’, Veldhoven, the Netherlands. The subjects were divided in three groups based on their self-reported laparoscopic experience: ‘novices’ without clinical experience but with understanding of the concept of laparoscopy, consisting of medical interns and 1st year residents. The ‘target’ group with more than 10 basic laparoscopic procedures performed but less than 20 advanced laparoscopic procedures. And the ‘expert’ group with extensive laparoscopic experience, including more than 20 advanced laparoscopic procedures performed, therefore consisting of residential surgeons in staff. The group setup was based on the results of the study by Botden et al. [20] showing assessment scores of novice laparoscopic intracorporeal suturing training that indicate a proficiency curve plateau observed from 15 sutures. Due to the non-medical intervention setup, no medical ethical approval was required.

### Equipment

**Fig. 1:**
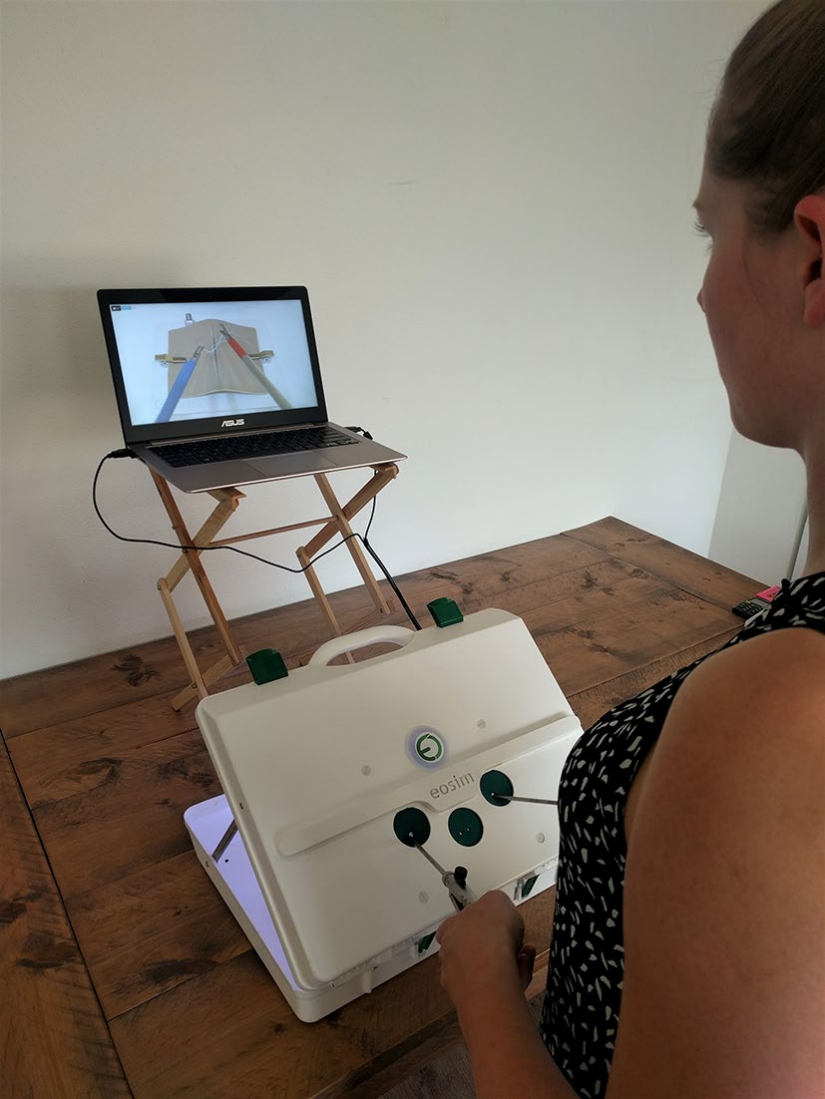
Set up of the eoSim as used in this study

In this study, the eoSim augmented reality laparoscopic simulator by Eosurgical ltd., Edinburgh, Scotland, United Kingdom was used in a standard setup (Fig. 1). This setup consisted of the eoSim laparoscopic case with an internal mounted high-definition camera and standard supplied equipment that consists of laparoscopic instruments, needle holders, suturing pad, thread transfer platform and a box with standard exercise equipment. A 15-in. laptop with the required specification as recommended by eoSurgical with the eoSurgical Surgtrac software installed. The tracking camera that is mounted in the case was connected to the laptop via USB 2.0. The Surgtrac software allowed for instrument tracking by means of the coloured markings on the instruments (right = red, left = blue). Placement of the laptop screen was adjusted to the proper height for every participant with the laparoscopic box being placed on a standard height table. The 30-mm curved needle braided thread sutures were used to perform the tasks.

### Tasks

Task 1 commenced with a video instruction, provided by the eoSurgical software. Two surgical knots had to be placed on the standard suturing pad in a horizontal plane (double wrap followed by two single wraps to create a secure surgeon’s knot). A standard length of 20 cm thread was used for each participant. If the thread of a suture was too short to reuse after being cut by the research assistant, a new suture would be placed on the suture pad.

Task 2 developed by the researchers to simulate abdominal wall suturing. This was simulated by using a cardboard panel to create an 80° plane on which a suture pad (Edustitch^®^, Breda, The Netherlands) was attached with the camera tilted upwards to simulate the difficult working angle (e.g. fixation of the stomach to the abdominal wall in gastrostomy placement). Each participant was given a total of three needles which had to be placed in the marked areas on the suturing pad with the tip of the needle being visible coming out of the suture pad again. In the case a needle was dropped, it was considered lost and the participant was required to grasp a new needle.

Task 3 simulated a running end-to-end anastomosis of an oesophagus or bowel anastomosis. This was performed with two cut out balloons as shown in Fig. 4, with eight dots around the edges on each side. Participants were required to transfer a needle through all the dots as if they were placing an anastomosis without knot tying, resulting in 16 transfers to complete the task. During the study, a high difficulty of the task was noticed for each experience group to complete the task. Therefore, a simplified version of the task was constructed as shown in Fig. 4. This simplified version consisted of four dots around the edge of each balloon to decrease the duration of the task. To take these changes in the task during the study into account, a subanalysis has been performed.

### Questionnaire

The questionnaire used in this study was previously used in other validation studies [21, 22]. The questionnaire consisted of two parts, in which the first part contained the informed consent, demographics including age, dexterity, previous simulator experience and the participant’s current laparoscopic experience. The laparoscopic experience was elaborated as current profession, years in surgical/gynaecologic/urologic training and number of basic and advanced laparoscopic procedures performed. Basic laparoscopic procedures were defined as non-suturing procedures, such as cholecystectomy and appendectomy. Advanced laparoscopic procedures were defined as procedures with intracorporeal suturing, such as gastric fundoplication, bariatric surgery or colon surgery. The second part of the questionnaire consisted of questions on the realism (regarding realistic response, grasper use, force feedback, supposed simulated task and tissue behaviour), didactic value (value to train novice surgeons, experienced surgeons and ability to assess the skills of a trainee) and usability (attractiveness, user friendliness and possibility of instruments selection) of the system. These were rated for each separate task, on a five-point-Likert scale. With ‘1’ resulting in strong disagreement, ‘3’ being the neutral opinion and ‘5’ resembling a strong agreement [23]. There also was an option to react ‘No answer’.

### Protocol

**Fig. 2:**
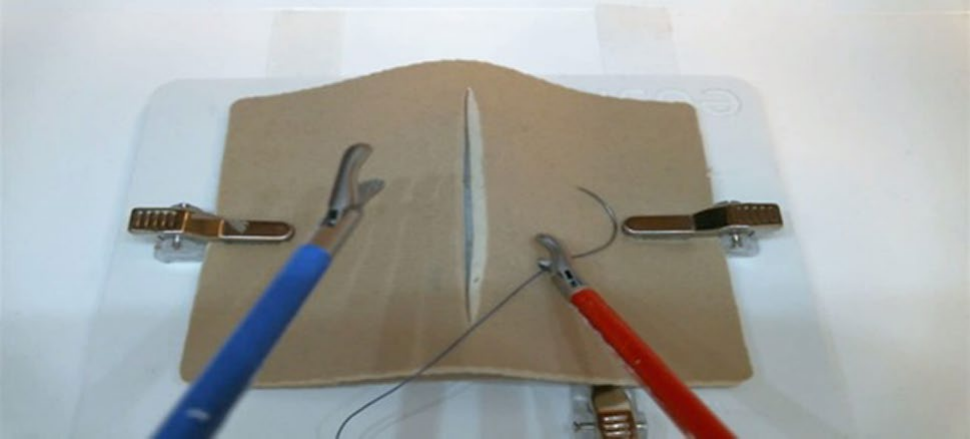
Screenshot of the vertical defect suturing (Task 1)

**Fig. 3:**
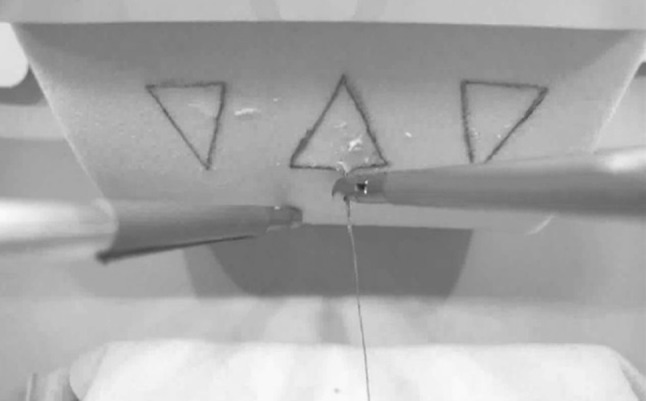
Screenshot of the up side down needle transfer (Task 2)

**Fig. 4:**
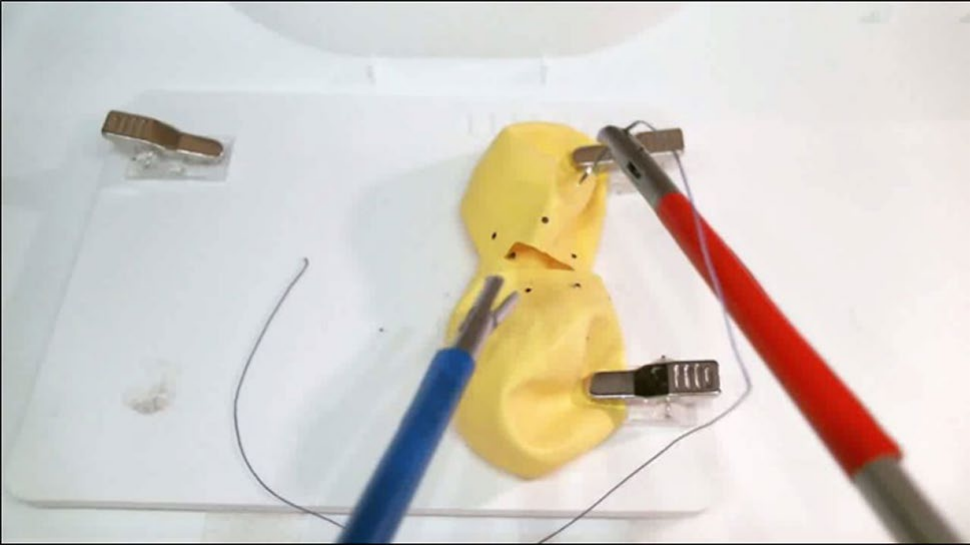
Screenshot of the anastomosis needle transfer (Task 3)

After completing the first part of the questionnaire, each participant received a short instruction on the functionality of the system and were allowed to practice each exercise once to get familiar with the system. Subsequently, the participants performed the intracorporeal suturing exercise (Task 1, Fig. 2), the upside down needle transfer task (Task 2, Fig. 3) and the anastomose needle transfer (Task 3, Fig. 4). All exercises had a maximum of 20 min to be completed. After completion of the exercises, participants completed the remainder of the questionnaire regarding their opinion on the realism, didactic value and usability of each performed task.

### Outcomes

The recorded parameters of the simulator consisted of time to complete the task (seconds) and specific parameters based on the tracking of the instruments tips, which were recorded for each instrument separately (left and right). These parameters were path length (centimetres), instrument tip off-screen (percentage), average speed (millimetre per second) and the working area (average distance between instruments in centimetres). Acceleration (millimetre per square second) and motion smoothness (millimetre per second to the third exponentiation) were also presented. These parameters are derivatives from the ‘speed’ parameter.

### Statistical analysis

Mean outcomes for face, content and construct validity were analysed for statistical differences between groups using a one-way ANOVA test. Regarding the face and content validity outcome ‘3’ represents ‘neutral’ on the questionnaire therefore, a mean of > 3.5 was considered valid. The analysis was performed using Statistical package for social sciences (SPSS) version 22 (IBM Corp., Armonk, NY). Outcome parameters from the eoSim and the results of the questionnaires were inserted in the database and compared using a one-way ANOVA test with Welch correction for unequal variance. Results with a *p*-value of < 0.05 considered a statistically significant difference. To specify the origin of a significant *p*-value in the ANOVA test, a post hoc analysis was performed.

## Results

### Demographics

As shown in Table 1, a total of 105 participants were included in this study, with 60 participants in the novice group, 31 intermediates and 14 experts. Due to the high demand of skill and available time, not all included participants were able to finish all Tasks or follow the same order. Therefore, the order of Tasks was switched to avoid bias of fatigue or lack of time. In order to prevent possible selection bias, all participants were included for analysis. From the total number of participants, 94 performed Task 1 (50 novices, 31 intermediates and 13 experts), 69 performed Task 2 (39 novices, 21 intermediates and 9 experts) and 45 performed Task 3 (31 novices, 6 intermediates and 8 experts). The novice groups consists of only interns and 10 junior postgraduate year (PGY) residents. The target group consisted of surgical residents (mainly PGY 4–5, *n* = 21. 68%). The expert groups consists of 13 specialized surgeons and one 6th year resident.

**Table 1:** Demographics of participants

	Novice (*n* = 60)	Target (*n* = 31)	Expert (*n* = 14)	Total (*n* = 105)
Mean age (SD)	25 (3.2)	34 (4.7)	43 (11.2)	30 (7.9)
Dexterity				
Right handed	88.1%	87.1%	92.3%	88.5%
Left handed	11.9%	9.7%	0.0%	9.6%
Ambidextrous	0.0%	3.2%	7.7%	1.9%
Education				
Intern	50	0	0	50
Postgraduate year (years)				
1	9	2	0	11
2	1	1	0	2
3	0	4	0	4
4	0	12	0	12
5	0	9	0	9
6	0	0	1	1
Specialized surgeon	0	3	13	16

*SD* standard deviation

### Face and content validity

#### Task 1: intracorporeal suturing

The overall mean scores of the realism (3.9), didactic value (4.0) and usability (4.2) show positive opinion scores without any significant differences (Table 2). Analysis of difference in opinion scores between the groups for individual questions did not show any statistical differences. The realism of behaviour of the tissue (Novice 3.4 Target 3.2 Expert 3.0) was scored lowest. The didactic value of this module to train novice laparoscopic surgeons (Novice 4.2 Target 4.2 Expert 4.1) was scored the highest.

**Table 2:** Mean opinion scores (SD) on realism, didactic value and usability of the three performed tasks on the eoSim

Intracorporeal suturing	Expert (*n* = 13)	Target (*n* = 31)	Novice (*n* = 50)	Total (*n* = 94)	*p*-value
Realism	3.9 (0.8)	3.9 (0.5)	3.9 (0.6)	3.9 (0.6)	0.953
Didactic value	4.0 (0.8)	4.0 (0.5)	3.9 (0.7)	4.0 (0.6)	0.573
Usability	4.0 (1.0)	4.2 (0.7)	4.2 (0.6)	4.2 (0.7)	0.618

Upside down needle transfer	Expert (*n* = 9)	Target (*n* = 21)	Novice (*n* = 39)	Total (*n* = 69)	
Realism	3.3 (1.2)	3.3 (0.9)	3.6 (0.6)	3.6 (0.6)	0.283^*^
Didactic value	2.6 (1.4)	3.4 (1.1)	3.7 (0.8)	3.4 (1.0)	0.082^*^
Usability	3.2 (1.3)	4.0 (0.8)	3.7 (0.8)	3.7 (0.9)	0.226

Anastomosis needle transfer	Expert (*n* = 8)	Target (*n* = 6)	Novice (*n* = 31)	Total (*n* = 45)	*p*-value
Realism	3.5 (0.9)	3.6 (0.9)	3.8 (0.6)	3.7 (0.7)	0.421
Didactic value	3.5 (0.8)	3.2 (1.1)	3.9 (0.6)	3.7 (0.8)	0.071
Usability	3.8 (1.4)	4.1 (0.8)	4.0 (0.7)	4.0 (0.8)	0.734

Differences in opinion scores were calculated with the ANOVA test. A *p*-value of < 0.05 was considered a significant difference

*SD* standard deviation

*p-value after Welch correction for unequal variances

#### Task 2: upside down needle transfer

The overall mean score of this self-made task on realism (3.6) didactic value (3.4) and usability (3.7) was slightly lower than Task 1 (Table 2). Analysis of the mean individual questions shows tissue realism (Novice 3.1 Target 3.0 Expert 2.0), and realism of the suture running through the tissue (Novice 3.2 Target 3.1 Expert 3.1) was scored the lowest. Realism of the on-screen response of the instruments received high scores (Novice 3.8 Target 3.5 Expert 4.1). Analysis of the difference in opinion scores between the groups for the individual questions, resulted a *p*-value of < 0.001 regarding the didactic value of the module to train novice laparoscopic surgeons (Novice 3.8 Target 3.3 Expert 2.0). Post hoc analysis shows a statistical difference between Experts versus Novices (*p* < 0.001) and Experts versus Target group (*p* = 0.033).

#### Task 3: anastomosis needle transfer

This task resulted in positive total mean scores on realism (3.7) didactic value (3.7) and usability (4.0 Table 2). The individual question analysis scored lowest means in realism of the tissue (Novice 3.2, Target 2.7, Expert 2.8). The highest scores were again found on the realism of the on-screen response (Novice 4.2, Target 4.0, Expert 4.4). Analysis of the difference in mean opinion scores resulted in a statistical difference on the didactic value of this module to train novice laparoscopic surgeons (Novice 4.1, Target 3.0, Expert 2.5. *p* = 0.002). Post hoc analysis results in a statistical difference only between Experts versus novices (*p* = 0.004).

### Construct validity

As depicted in Table 3, there are significant differences between the groups in time (*p* < 0.001), distance (*p* < 0.001) and acceleration (*p* = 0.042) for Task 1. Task 2 shows significant differences in time (*p* < 0.001), distance (*p* = 0.003), instruments off-screen (*p* < 0.001) and working area (*p* < 0.001). Task 3, however, shows no significant differences in the system outcome parameters between the groups, although experts were faster compared to novices (764 versus 1123 s, *p* = 0.092) and had travelled a shorter distance with their instruments (13.0 cm versus 22.8 cm, *p* = 0.107). Due to the difficulty to complete the 16 stitches anastomosis task, a subanalysis was performed in which the anastomosis required only eight sutures. This subanalysis shows the difference in the completion of the task between novices and experts for the 16 sutures (38% versus 0%, *p* = 0.003) and eight sutures (100% versus 35%, *p* = 0.003). Other relevant factors were the number of completed sutures (13.6 versus 5.9, *p* < 0.001), time per suture (57.6 versus 100.5, *p* < 0.001) and instrument distance per suture (1.04 versus 4.29, *p* < 0.001) between the expert and novice group.

**Table 3:** Construct validity of the eoSim

Intracorporeal suturing	Expert (*n* = 13)	Target (*n* = 31)	Novice (*n* = 50)	*p*-value
Time (s)	339 (114)	607 (229)	1124 (578)	< 0.001^*^
Distance (m)	8.1 (3.5)	15.6 (6.3)	21.7 (15.3)	< 0.001^*^
Off-screen (%)	6.5 (4.8)	9.9 (7.4)	8.2 (7.0)	0.302
Speed (mm/s)	2.67 (0.69)	4.42 (2.62)	7.52 (29.31)	0.698
Acceleration (mm/s^2^)	1.27 (0.49)	2.15 (1.08)	1.67 (1.23)	*0.042*
Smoothness (mm/s^3^)	0.008 (0.009)	0.024 (0.039)	0.011 (0.018)	0.088^*^
Working area (cm)	5.72 (0.96)	5.14 (0.94)	5.41 (1.17)	0.242

Upside down needle transfer	Expert (*n* = 9)	Target (*n* = 21)	Novice (*n* = 39)	
Time (s)	478 (211)	519 (234)	897 (288)	< *0.001*
Distance (m)	18.8 (12.3)	13.4 (9.4)	26.6 (16.3)	*0.003*
Off-screen (%)	24.5 (11.5)	50.5 (20.6)	45.5 (37.4)	< 0.001^*^
Speed (mm/s)	5.85 (3.30)	7.01 (3.05)	6.38 (3.03)	0.590
Acceleration (mm/s^2^)	2.08 (1.08)	1.70 (0.94)	1.91 (1.09)	0.606
Smoothness (mm/s^3^)	0.019 (0.025)	0.011 (0.014)	0.017 (0.051)	0.802
Working area (cm)	7.38 (2.59)	3.16 (2.26)	4.77 (2.85)	< *0.001*

Anastomosis needle transfer	Expert (*n* = 8)	Target (*n* = 6)	Novice (*n* = 31)	*p*-value
Time (s)	764 (445)	934 (290)	1123 (224)	0.092^*^
Distance (m)	13.0 (8.6)	22.6 (11.3)	22.8 (12.2)	0.107
Off-screen (%)	7.8 (15.2)	10.6 (15.0)	3.4 (6.1)	0.465^*^
Speed (mm/s)	2.26 (1.17)	3.26 (2.04)	2.70 (1.40)	0.441
Acceleration (mm/s^2^)	1.11 (0.52)	1.38 (0.68)	1.42 (0.70)	0.523
Smoothness (mm/s^3^)	0.003 (0.007)	0.002 (0.004)	0.002 (0.007)	0.963
Working area (cm)	4.82 (1.63)	3.59 (1.79)	4.65 (1.00)	0.129
Total of number stitches	13.6 (3.0)	10.0 (3.8)	5.9 (2.5)	< *0.001*
Distance per stitch (m)	1.04 (0.79)	2.29 (0.91)	4.29 (2.85)	< 0.001^*^
Time per stitch (s)	57.6 (33.0)	100.5 (39.0)	219.5 (112.6)	< 0.001^*^
Completed 16 stitches	3 (38%)	1 (17%)	0 (0%)	*0.003* ^**^
Completed 8 stitches	8 (100%)	4 (67%)	11 (35%)	*0.003* ^**^

Data in this table represent the mean value (standard deviation). Statistical differences were calculated with the ANOVA test. A *p*-value < 0.05 (displayed in italic) was considered significant

*p-value after Welch correction for unequal variances

**p-value after Chi-square test

To determine between which groups there was a significant difference, a post hoc analysis was performed as shown in Table 4. There was a significant difference between all the groups for ‘time’ and ‘distance’ in Task 1. However, for ‘acceleration’, the significant *p*-value of 0.042 of the initial analysis could not be proven significant between groups. Regarding Task 2, the ‘time’ to complete the task is significantly different between experts versus novices and novices versus target group (*p* < 0.001). It was not significantly different between novices and target although, the target group performed Task 2 in less time (519 s versus 897 s, *p* = 0.885). The parameter ‘distance’ is significantly different for intermediates versus novices (*p* < 0.001) indicating more instrument movement in the novice group. This could not be shown for the expert versus novice group. However, despite the shorter instrument path of the experts (18.8 m versus 26.6 m, *p* = 0.267), ‘working area’ and ‘off-screen’ show construct validity for experts versus novice (*p*-values 0.029 and 0.017, respectively) and experts versus target group (both *p* < 0.001). Task 3 shows significant differences between experts versus novices and novice versus target group for ‘number of stitches completed’ (*p* < 0.001 and 0.005, respectively), ‘time per stitch’ (both *p* < 0.001), ‘distance per stitch’ (*p* < 0.001 and 0.010, respectively) and ‘completion 16 stitches’ (*p* < 0.001 and 0.019, respectively). ‘Completion of eight stitches’ only shown significant differences for experts versus novices (*p* < 0.001).

**Table 4:** Post hoc ANOVA analysis of significant results

*p*-values	Parameter	Expert versus novice		Expert versus intermediate	Intermediate versus novice
Task 1: Intracorporeal suturing	Time		< *0.001*	< *0.001*	< *0.001*
	Distance		< *0.001*	< *0.001*	*0.037*
	Acceleration		0.769	0.059	0.188
	Smoothness		0.558	0.080	0.213
	Off screen		1.000	0.435	0.827
	Working area		0.770	0.183	0.830
Task 2: Upside down needle transfer	Time		< *0.001*	0.885	< *0.001*
	Distance		0.267	0.429	< *0.001*
	Off screen		*0.017*	< *0.001*	0.736
	Working area		*0.029*	< *0.001*	0.079
Task 3: Anastomosis needle transfer	Time		0.128	0.671	0.347
	Number of stitches		< *0.001*	0.057	*0.005*
	Time per stitch		< *0.001*	0.126	< *0.001*
	Distance per stitch		< *0.001*	0.054	*0.010*
	Completed 16 stitches^*^		< *0.001*	0.392	*0.019*
	Completed 8 stitches^*^		< *0.001*	0.078	0.138

After testing for homogeneity of variance either Bonferroni correction (equal variances) or the Games-Howell correction (unequal variance) was used

*p-value after Chi-square test. A *p*-value of < 0.05 (displayed in italic) was considered significant

## Discussion

With the growing need for accessible and affordable minimal invasive simulators for the training of surgeons, the eoSim laparoscopic simulator is able to fulfil this need. The eoSim laparoscopic simulator offers the instrument tracking capabilities of a virtual reality system, but is combined with the natural haptic feedback of traditional inanimate training boxes. This study demonstrates the fundamental validity of the eoSim regarding the construct, content and face validity. The system is capable of differentiating the between expert and novice users on multiple outcome parameters regarding advanced suturing tasks. Its versatility allows it to be a potential valid training tool to train basic tasks up to complex suturing tasks or even personal adjusted tasks as done in Task 2 in this study to simulate upside-down suturing.

With the mobile case setup only requiring a laptop it is accessible to train in almost every location, by anyone. This could be beneficial for the clinical setting in which surgeons can practice difficult tasks in a safe setting and refresh their skills before starting a complex surgical procedure. Subsequently, this can affect improvement of the quality of sutures and anastomoses in the patient and reduce complications.

Previous validation studies of the eoSim laparoscopic simulator have been performed [8–10], but none of those articles were based on the current version of the eoSim or regarding basic laparoscopy. Hennessey et al. concluded a significant construct and concurrent validity of the standard suturing task (Task 1 of this study). However, no face validity was demonstrated. Also, an overall scoring rate from 0 to 100% was used for the outcomes of the simulator, without a calculation of the separate outcomes, making a comparison with the outcomes from this study difficult (17). Retrosi et al. did validate the current version of the eoSim, regarding a similar suturing task, but was adjusted for paediatric surgery (16). They concluded a construct and concurrent validity of the eoSim for the suturing task in a paediatric simulation. However, construct validity for the parameters smoothness, acceleration and speed as demonstrated in that study was not observed in the current study. This could be due to multiple factors, including the difference in population size (28 participants versus 104 participants in the current study), newer software version with improved tracking or a difference in task protocol. We assessed both the face, content and construct validity of the suturing tasks on the eoSim with a larger population including the target group, in a multicentre setting, compared to previous studies.

Outliers in the results of this study regarding face validity are shown in Task 2. The didactic value of the upside-down task to train novice surgeons scored relatively low by the expert group (mean 2.6), which can be due to the high difficulty of the task. The upside down needle transfer was not designed for novice surgeons and therefore it is an expected outcome on this question. Also, the experienced surgeons struggled with this task as well, which could bias their opinion on this task in general. The construct validity has been determined for Task 2, because it has the capability to differentiate between experts and novices on the level of time, off-screen and working area. However, for the anastomosis needle transfer task these results were not reached. This could be due to the lower number of participants, because this task was a time-consuming exercise and demanded a very high level of laparoscopic skill. The parameters ‘time’ and ‘distance’, however, did have a relevant difference between the experts and novices (means 764 s and 13.0 m versus 1123 s and 22.8 m, *p* = 0.092 and *p* = 0.107).

To determine the differences between each group, a post hoc analysis was performed which shows significant differences between novices and experts for ‘time’ and ‘distance’ in Task 1, as well as ‘time’, ‘working area’ and ‘off-screen’ in Task 2. Therefore, the construct validity on these parameters has been established. Construct validity could not be established on all separate parameters produced by the simulator. It is, however, important to assess the clinical meaningful parameters for this construct validity. Time, for example, shows how fast a task is completed, but it does not provide any information on how a task has been performed. Regarding the technical performance and safety, the parameters ‘off-screen’, ‘working area’, ‘distance’, ‘acceleration’ and ‘speed’ are more relevant indication of what the participant has done with its instruments movements and in the surgical area where that happened. It is also important to notice that ‘acceleration’ (mm/s^2^) and ‘smoothness’ (mm/s^3^) are calculated from the parameters ‘speed’ (mm/s), therefore they should not be taken into account as primary parameters to base the construct validity on. However, it would be meaningful to have a construct validity established on multiple parameters to give a complete assessment and possible proficiency score of the performed task. Thus far these calculated parameters should not be used for assessment purposes.

### Limitations

There were some limitations in this study that need to be considered. The tasks that were validated were difficult and complex, which led to participants getting frustrated. This could have a negative influence on the opinion of the eoSim, affecting the face validity. Intracorporeal suturing is a complex task to perform and often replaced by methods like self-locking sutures or staples to reduce operating time and workload [24]. Only a small group of surgeons were adequately trained to meet the criteria of > 20 advanced laparoscopic procedures with intracorporeal suturing and therefore qualified as experts, making it difficult to include a sufficient number of participants in this category. Due to the switch of a simpler anastomosis for Task 3, there could be a bias regarding the parameters of the system, because the participants who performed and completed the eight sutures anastomosis within the time limit would have lower parametric scores and therefore affect the average group score. To account for this possible bias, a subanalysis per suture was performed.

During the execution of the tasks, the participants also could notice the changing focus of the installed webcam which was on either the suture pad or the instruments but could not be manually adjusted to the user’s preference. This could cause frustration to the participants and affect their opinion and performance.

## Conclusion

In this study, the eoSim was regarded as a useful tool to train complex suturing tasks by the expert, target and novice participants. Also, the eoSim was capable to determine the difference between expertise levels in terms of ‘time’ and ‘distance travelled by the instruments’, on these complex tasks, demonstrating the construct validity of these parameters. Moreover, the relative low costs, easy transferability of this simulator and option of introducing adapted tasks for the training of a specific type of surgery, make it an attractive training tool for any minimal invasive surgery specialism. With these results, the eoSim is a potential valid tool for the training and assessment of advanced laparoscopic suturing.
